# Flavobacteria consume nitrous oxide produced by partial denitrifiers in coastal sediments

**DOI:** 10.1093/ismejo/wrag186

**Published:** 2026-07-14

**Authors:** Thanh Nguyen-Dinh, Tess F Hutchinson, Francesco Ricci, Hanif Prayitno, Luis Jimenez, Vera Eate, Pok Man Leung, Rachael Lappan, Sukhwan Yoon, Wei Wen Wong, Perran L M Cook, Chris Greening

**Affiliations:** Department of Microbiology, Biomedicine Discovery Institute, Monash University, Clayton 3168, VIC, Australia; Department of Microbiology, Biomedicine Discovery Institute, Monash University, Clayton 3168, VIC, Australia; Department of Microbiology, Biomedicine Discovery Institute, Monash University, Clayton 3168, VIC, Australia; Water Studies, School of Chemistry, Monash University, Clayton 3800, VIC, Australia; Department of Microbiology, Biomedicine Discovery Institute, Monash University, Clayton 3168, VIC, Australia; Water Studies, School of Chemistry, Monash University, Clayton 3800, VIC, Australia; Department of Microbiology, Biomedicine Discovery Institute, Monash University, Clayton 3168, VIC, Australia; Department of Microbiology, Biomedicine Discovery Institute, Monash University, Clayton 3168, VIC, Australia; Department of Civil and Environmental Engineering, KAIST, Daejeon 34141, South Korea; Water Studies, School of Chemistry, Monash University, Clayton 3800, VIC, Australia; Water Studies, School of Chemistry, Monash University, Clayton 3800, VIC, Australia; Department of Microbiology, Biomedicine Discovery Institute, Monash University, Clayton 3168, VIC, Australia

**Keywords:** nitrous oxide, permeable sediments, partial denitrifiers, low affinity clade II nosZ, Flavobacteria

## Abstract

Nearly one-fifth of global emissions of the potent greenhouse gas nitrous oxide (N_2_O) originate from the ocean, particularly from nutrient-polluted coastal regions. Permeable (sandy) sediments, which cover half of the continental shelf worldwide, are potential sources of N_2_O due to increasing nutrient inputs from urbanization and agriculture. Yet, the microbial processes determining N_2_O emissions in these dynamic and unique ecosystems remain understudied. Here, we combined environmental measurements, bacterial cultivation, and genomic analyses to understand the microbes and processes controlling N_2_O cycling in permeable sediments from Port Phillip Bay (Australia). We established a genomic resource comprising 249 metagenome-assembled genomes and 95 new isolate genomes. Genome-based metabolic reconstructions and culture-based gas measurements revealed that diverse bacteria in these sediments produce N_2_O through incomplete denitrification pathways. However, these bacteria co-occurred with highly abundant clade II N_2_O-reducing bacteria from the *Flavobacteriaceae* family. Kinetic profiling showed that both clade II *nosZ* flavobacterial isolates and whole sand communities exhibited a low apparent affinity for N_2_O under the tested experimental conditions, expanding the currently limited kinetic data available for N_2_O reducing microorganisms from coastal permeable sediments, including flavobacterial clade II N_2_O reducers. Collectively, these findings indicate that abundant N₂O reducing communities can substantially consume N_2_O within permeable sediments, thus limiting N_2_O accumulation despite active N_2_O production. Together with previous hydrodynamic models predicting low N_2_O release from permeable sediments, our results highlight the important role of specialized microbial communities in regulating N_2_O cycling under increasing nutrient pollution.

## Introduction

Nitrous oxide (N_2_O) is a long-lived atmospheric trace gas contributing to stratospheric ozone depletion and climate change [1]. With a warming potential ~300 times higher than that of carbon dioxide on a 100-year time scale, N_2_O is the third largest contributor (6.4%) to total greenhouse gas emissions after CO_2_ and CH_4_ [2, 3]. Up to 57% of the total N_2_O emissions come from natural sources, in which marine ecosystems contribute to nearly half of such emissions at 2–7 Tg N year^−1^ [4]. Estuarine and coastal zones function as a natural biofilter, removing excess nutrients at the interface between land and ocean [5]. However, increasing anthropogenic activities, such as nutrient loading, aquaculture, and fertilizer use, are inputting nitrogen to these ecosystems, which in turn results in widespread eutrophication and exacerbates N_2_O emissions [4, 6–9]. N_2_O emissions from coastal environments remain insufficiently understood and poorly quantified in global N_2_O budgets due to complex N_2_O production pathways and high spatiotemporal variability [7, 10, 11].

Permeable sediments (i.e. sands and gravels) dominate coastal regions, covering nearly half of the continental shelf worldwide [12]. These sediments are subject to highly turbulent hydrodynamic conditions that vary across space and time, as opposed to fine-grained impermeable (cohesive) sediments [13–15]. Advective porewater flows drive continuous exchange of dissolved solids and gases, suspended particles, and microorganisms between the sediment and overlying water column, making redox conditions in the sediments highly variable over short distances and timescales [13–15]. With microbial abundance of ~10^9^ cells cm^−3^ sediments [16], sands affect N_2_O emissions in coastal regions due to enhanced microbial nitrogen cycling linked to anthropogenic nitrogen inputs. Nitrification and denitrification are the two main microbial processes that control the N_2_O budget in aquatic environments [17, 18]. In well-oxygenated waters, N_2_O can be released by ammonia-oxidizing bacteria (AOB) and archaea (AOA) as a byproduct of ammonia oxidation to nitrite (NO_2_^−^) (i.e. the first step of nitrification) [9, 18]. By contrast, N_2_O is produced as an intermediate during denitrification of nitrate (NO_3_^−^) or NO_2_^−^ under oxygen-depleted conditions [17, 19]. Many denitrifiers lack the genetic capacity for N_2_O reduction, resulting in N_2_O released as the final product instead of inert dinitrogen gas (N_2_) [20, 21]. Furthermore, abiotic processes, such as (photo)-chemodenitrification, can also contribute to the production of N_2_O in marine waters and sediments [22, 23].

Despite several processes leading to N_2_O production, the major biological sink of this gas is its reduction to dinitrogen gas (N_2_) by microorganisms expressing the periplasmic, copper-containing enzyme N_2_O reductase (NosZ) [24, 25]. Phylogenetic analyses resolve the enzyme into clade I (NosZI), clade II (NosZII), and the recently defined clade III (NosZIII) [26–28]. NosZI is often associated with complete denitrifiers. In contrast, NosZII is found in much more diverse microbial taxa that typically lack key denitrification enzymes (i.e. NirK and NirS), suggesting that N_2_O serves as an electron acceptor even for non-denitrifiers [27–29]; e.g. some non-denitrifying bacteria encoding the NosZII, including the acidophilic bacterium *Desulfosporosinus nitrosoreducens* [30] and methanotrophs *Methylocella tundrae* and *Methylacidiphilum caldifontis* [31], reduce N_2_O to conserve energy through anaerobic respiration, while others use it to dispose excess electrons during transient anoxia [32]. Beyond these two clades, a recent study discovered a widespread and active clade III lactonase-type NosZ that is divergent in sequence but similar in structure to NosZI and NosZII [26]. These findings have expanded our understanding on the diversity of N_2_O reducers and provide opportunities for mitigating N_2_O emissions.

Given the global extent of permeable sediments, any imbalances between N_2_O production and consumption can greatly impact marine N_2_O budgets. Although there is evidence that these sediments are a potential source of N_2_O [33–37], recent hydrodynamic-biogeochemical models suggest that their contribution to coastal N_2_O emissions are relatively minor [38], highlighting uncertainties in the N_2_O budget of these sediments. Furthermore, current understanding of the microbial basis of N_2_O cycling within permeable sediments is largely limited to community surveys [34], with little functional and ecophysiological characterization. In this study, we addressed these knowledge gaps by investigating the functional diversity and capacity of N_2_O-cycling microorganisms in sandy sediments from moderate to high nutrient-impacted beaches in Port Phillip Bay, Australia. We hypothesized that permeable sediments host metabolically diverse and functionally coupled N_2_O-producing and N_2_O-consuming microorganisms whose interactions regulate net N_2_O fluxes. To test this hypothesis, we integrated bacterial cultivation, metagenomic analyses, and biogeochemical assays to provide a newly compiled genome collection of microorganisms inhabiting permeable sediments and assess their role in N_2_O cycling. Altogether, we demonstrate that diverse N_2_O producers and consumers are present and active in coastal sediments, with their tight coupling buffering the N_2_O emissions from these sediments. These findings provide microbial and ecophysiological evidence supporting hydrodynamic models [38] that coastal N_2_O emissions may be overestimated and offer mechanistic microbial insights that can be incorporated into future predictions of coastal N_2_O fluxes.

## Materials and methods

### Sampling and *in situ* N_2_O-level measurements

Different sampling campaigns were conducted at St. Kilda Beach (37.863159°S, 144.971026°E) and Werribee Beach (37.58132°S, 144.42112°E) between 2022 and 2024 for different purposes: (i) bacterial isolation (02/06/2022, 03/08/2022, 19/10/2022, and 11/01/2023), (ii) slurry incubations (10/05/2024 for St. Kilda samples; 23/09/2024 and 20/08/2025 for Werribee samples), and (iii) *in situ* N_2_O concentrations (24/05/2024 and 05/11/2024 for Werribee and 15/05/2024, 01/10/2024, and 18/11/2024 for St. Kilda). Subtidal surface sediment (0–5 cm deep) was collected in sterile 50-ml Falcon tubes and were immediately transported to the laboratory and stored at 4°C overnight before experiments. The sediments for metagenomic sequencing were also subsampled, flash-frozen in liquid nitrogen, and stored at −20°C until further analysis. Seawater samples were collected for slurry incubations and were filtered (0.22 μm) to remove most pelagic species.

*In situ* N_2_O levels were measured from porewater samples collected with a piezometer inserted to depths of 0, 10, 20, and 30 cm below the sediment surface. The piezometer was connected to a syringe via a rubber tube and over-filled into 12-ml Exetainer vials. Three replicates were collected, each spaced 2 m apart. Samples were preserved by adding 50 μl saturated HgCl_2_ and stored at room temperature. For dissolved N_2_O concentration measurement, a 4-ml helium headspace was introduced into each 12.5-ml Exetainer vial, shaken, and equilibrated at room temperature before being analysed by gas chromatography described below.

### Slurry incubations

Sediment experiments were conducted in order to determine N_2_O consumption capability of the sediment community. Slurries were prepared with 5 g of wet sediment and 50 ml filtered seawater in 120-ml serum vials before sealing with a butyl rubber stopper and Wheaton closed-top seals. Serum vials were purged with helium (He) gas for 10 min with vigorous shaking every 5 min to exclude O_2_ and create anoxic conditions. The headspace of vials was amended with N_2_O (Air Liquide Australia Limited, Victoria, Australia) to give initial mixing ratios of ~10 or 1000 ppmv, which correspond to equilibrium aqueous concentrations of 0.8 and 108 μM, respectively, calculated as previously described [39]. Before measurement, 2 ml of He was injected into the headspace, then 2 ml of sample was taken from the headspace and injected into a He pre-purged 3 ml Exetainer. Samples were analysed using a VICI Trace Gas Analyser with a limit of detection of 0.05 ppmv.

### Metagenomic sequencing and assembly

Environmental DNA was extracted from six sediment samples collected at St. Kilda and Werribee beaches using the PowerSoil DNA kit (QIAGEN) following the manufacturer’s instructions. Extractions included a sample-free negative control. Samples were sequenced on a NovaSeqX Plus System (Illumina) (2 × 150 bp) at Monash Genomics & Bioinformatics Platform (Monash University). Sequencing yielded an average of 44 021 402 read pairs per sample, with 1052 read pairs sequenced in the negative control. The metagenomes were processed using the Metaphor workflow [40]. Specifically, the metagenomes were quality filtered with fastp [41] and co-assembled using MEGAHIT v1.2.9 [42] with default settings. Contigs <1000 bp were discarded. The assembled contigs were binned with Vamb v4.1.3 [43], MetaBAT v2.12.1 [44], and CONCOCT v1.1.0 [45]. The three bin sets were subsequently refined using DAS Tool v1.1.6 [46], combined with MAGs previously obtained from St. Kilda permeable sediments [47, 48] and dereplicated with dRep v3.4.2 [49] using the parameters -comp 50 -con 10. The completeness, contamination, and heterogeneity of the bins were assessed using CheckM2 [50]. The presence of rRNAs and quantification of tRNAs in each bin were estimated by MAGqual v0.3.0 [51]. This information was used to set the quality thresholds for bins according to MIMAG standards [52]: high quality (>90% completeness, <5% contamination, rRNA genes and ≥18 tRNAs) and medium quality (>50% completeness and <10% contamination). These high-quality and medium-quality bins were retained for further processing, which were termed metagenome-assembled genomes (MAGs). MAG taxonomy was determined using the Genome Taxonomy Database Release R226 [53] via GTDB-Tk v2.3.2 [54]. CoverM [55] with the option ‘genome’ was used to calculate the relative abundance of each MAG in each sample. Community composition was profiled from quality-filtered short reads by assembling and classifying 16S rRNA genes with PhyloFlash v3.4 [56].

### Bacterial isolation and whole-genome analysis

Sediment samples for bacterial isolation were collected at St. Kilda Beach in three sampling campaigns (02/06/2022, 03/08/2022, and 11/01/2023) and at Werribee Beach (19/10/2022). Approximately 1 g wet weight of the surface sediment (0–5 cm deep) was dispersed in 9 ml of sterilized 1× phosphate-buffered saline (PBS), vortexed vigorously for 3 min and sonicated for another 3 min. The resulting slurry was serially diluted with 1× PBS and stored in cryovials at −80°C with 25% glycerol for further isolation if needed. Aliquots of 100 μl undiluted and serially diluted slurry were spread on Marine Agar 2216 (Difco), modified Marine Agar 2216E, and Marine R2A Agar plates (ingredients in Table S1) and incubated aerobically at 30°C for at least 1 week until most morphologically different colonies appeared on the plates. A series of enrichments were also performed using 1 mM NaNO_3_ or NaNO_2_ as electron acceptors and either 1 mM sodium acetate, 1 mM glucose, 0.002 g/L starch, or 0.5 g/L yeast extract as electron donors. Approximately 5 g of sediments was suspended in 50 ml of a bicarbonate-buffered minimal saltwater medium (MSW) (Table S1) in 120-ml serum bottles and purged with N_2_ for 3 min to create anoxic conditions. Enrichments were transferred to fresh medium when NO_3_^−^/NO_2_^−^ was all consumed, using 10% inoculum (v/v). After the third transfer, the slurries were serially diluted, isolated by streaking on Marine Agar 2216 or MSW agar, and incubated at the same conditions as mentioned above. Bacterial stocks were preserved at −80°C in 25% glycerol for long-term storage. For isolate identification, DNA was extracted by heating 30 μl of colony suspension at 95°C for 15 min and immediately frozen at −20°C and the 16S rRNA gene was amplified using the universal eubacterial primers 27F (5′-AGA GTT TGA TCC TGG CTC AG-3′) and 806rB (5′-GGA CTA CNV GGG TWT CTA AT-3′) or 1492R (5′-GGT ACC TTG TTA CGA CTT-3′). Purified PCR products were Sanger sequenced (Monash Genomics & Bioinformatics Platform, Victoria, Australia), and the obtained sequences were compared with those available in the GenBank database using BLAST on NCBI (National Center for Biotechnology Information) website.

A total of 95 bacterial isolates from permeable sediments collected in St. Kilda and Werribee beaches were selected for whole genome sequencing. The genomic DNA of isolates was extracted using the Genomic DNA Kit (Meridian Bioscience) following the manufacturer’s instructions. Samples were sequenced on a NovaSeq 6000 System (Illumina) at GENEWIZ (AZENTA Life Sciences). Raw sequences were quality filtered with the BBTools suite v.38.81 (https://sourceforge.net/projects/bbmap/), using BBDuk to remove the 151st base, trim adapters, filter PhiX reads, trim the 3′ end at a quality threshold of 15, and discard reads below 50 bp in length. Processed short reads were assembled using Unicycler v.0.4.7 [57]. The assembled genome quality was assessed with CheckM2 [50], and taxonomic classification of each isolate was performed using GTDB-tk v2.1.1 [54]. To estimate the relative abundance of the isolates in the sediments, quality-filtered metagenomic short reads were mapped against each isolate genome using CoverM, as described above.

### Metabolic annotation

Open reading frames (ORFs) were predicted using Prodigal v2.6.3 [58] for isolate whole genome and MAGs, then annotated using DIAMOND v2.0.15 [59] for alignment against a custom set of 51 metabolic marker protein databases (10.26180/c.5230745) covering the major pathways for aerobic and anaerobic respiration, energy conservation from organic and inorganic compounds, carbon fixation, nitrogen fixation, and phototrophy. The database was supplemented with the recently described clade III *nosZ* sequences [26] to ensure comprehensive coverage of the NosZ diversity. Gene hits were filtered by a minimum percentage identity score by protein: 80% (PsaA), 75% (HbsT), 70% (PsbA, IsoA, AtpA, YgfK, and ARO), 60% (CoxL, MmoX, AmoA, NxrA, RbcL, NuoF, [FeFe]-hydrogenases, and NiFe Group 4 hydrogenases), or 50% (all other genes). To determine gene abundance across the whole community, a similar approach was carried out to align the quality-filtered metagenomic short reads to the database. The number of aligned reads for each gene were normalized to reads per kilobase million mapped reads (RPKM) and then divided by the mean abundance of 14 universal single-copy ribosomal marker genes (in RPKM, obtained from the SingleM v0.13.2 package) [60]. This normalization provides a measure of average gene copies per genome.

### Phylogenetic analysis

To understand the distribution and diversity of microorganisms in marine permeable sediments involved in the nitrogen cycling and N_2_O emission, a phylogenomic tree was constructed from 95 bacterial isolate whole genomes and 249 MAGs. Genomes were aligned based on the AR53/BAC120 marker set of GTDB-Tk (v2.4.0) using the ‘align’ option and the tree was generated by maximum-likelihood estimation with the ultrafast bootstrap value of 1000 and model LG + F + I + G4 using IQ-TREE [61]. The full phylogenomic tree is provided in Fig. S2. For visualization in the main text, a representative subset of genomes was selected to capture major phylogenetic groups while reducing redundancy. A separate maximum-likelihood tree was reconstructed for this reduced dataset using IQ-TREE under the LG + F + I + G4 model with 1000 ultrafast bootstrap replicates. In addition, we also analysed the diversity of *nosZ* genes present in permeable sediments. To do so, we retrieved the NosZ amino acid sequences derived from unbinned and binned contigs and isolate genomes. Any sequences <300 amino acids were excluded. We also included the NosZ amino acid sequences from the Greening Lab database (10.26180/c.5230745) and clade III NosZ sequences from [26] as reference. Sequences were aligned using MAFFT v7.526 [62] and trimmed with TrimAL 1.2rev59 [63] with default settings. Maximum-likelihood trees were constructed with the ultrafast bootstrap value of 1000 and model Q.pfam+I + G4 using IQ-TREE [61]. The tree was visualized and midpoint rooted using the Interactive Tree of Life (iTOL) [64].

### Isolate physiological characterization

The partial denitrification phenotype was tested in two bacterial isolates (i.e. *Antarctobacter* sp. and *Sedimentitalea* sp.). The N_2_O-reducing phenotype was tested in two clade II *nosZ* organisms (i.e. *Olleya marilimosa* and *Winogradskyella* sp.) and two clade I *nosZ* organisms (i.e. *Marinobacter adherens* and *Oceanimonas* sp.). The isolates were selected to represent the phylogenetic diversity of target denitrifying and *nosZ*-containing lineages and for reliable laboratory growth, rather than their relative abundance in metagenomic datasets. All cultures were grown overnight in Difco 2216 Marine Broth medium under oxic conditions. Overnight cultures were transferred to 50 ml of fresh media so that the starting optical density (OD_600_) reached 0.03. Oxic treatment remained as unamended air while anoxic treatments were purged with He to exclude O_2_ as described above. For partial denitrification phenotype analysis, cultures were amended with either 1 mM NaNO_3_ or 0.1 mM NaNO_2_ and the concentrations of NO_x_ (NO_3_^−^ + NO_2_^−^) and NO_2_^−^ were determined spectrophotometrically on a Lachat Quickchem 8000 following the procedures in Standard Methods for Water and Wastewater [65]. Headspace gas (2 ml) was sampled into He purged 12-ml Exetainers to monitor N_2_O production. For N_2_O reduction phenotype analysis, the headspace of anoxic vials was amended with N_2_O as the main electron acceptor to give initial mixing ratios of ~100 and 1000 ppmv. Vials were placed on an orbital shaker (150 rpm) at 30°C for 24 h. To monitor N_2_O concentration changes, 2 ml of headspace gas was sampled into He purged 3-ml Exetainers.

### Kinetic analysis

Rates of N_2_O consumption by *O. marilimosa*, *Winogradskyella* sp., *M. adherens*, and *Oceanimonas* sp. and by whole-sediment communities from the two studied sites were measured. For axenic cultures, overnight-grown cells under oxic conditions (OD_600_ of 3.0 to 4.0) were inoculated into 50 ml fresh Difco Marine Broth medium in 120-ml serum vials to reach an initial OD_6oo_ of 0.03 then vials were sealed with butyl rubber stoppers. Similarly, slurry was prepared with 5 g of wet sediment and 50 ml filtered seawater in 120-ml serum vials and sealed with butyl rubber stoppers. Vials were shifted directly from oxic to anoxic condition by purging with He gas to evaluate the immediate capacity of isolates or microbial community to reduce N_2_O following this transition, reflecting the rapid redox fluctuations characteristic of permeable sediments. Accordingly, the assay captures N₂O reduction potential immediately after the oxic–anoxic transition rather than steady-state kinetics under prolonged anoxic growth. Triplicate cultures or slurries were incubated with different N_2_O concentrations in the headspace ranging from 5 to 1000 ppmv. Headspace N_2_O concentrations were quantified by gas chromatography at different time intervals. The final N_2_O concentration in the liquid phase was corrected and calculated as previously described [39]. Reaction rates of N_2_O consumption were determined from the slopes of linear regression lines fitted to N_2_O concentration measurements over time. Reaction rates were normalized either by wet sediment weight for community kinetics or to total cell protein content for isolate cultures. Protein concentrations of the pre-dilution aerobic cultures were quantified using BCA protein assay (Sigma-Aldrich) and used to estimate protein content in the assay cultures by scaling according to the dilution factor used to initial OD_600_ of ~0.03. Based on preliminary experiments showing no measurable growth under anoxic conditions, biomass was assumed to remain constant during the kinetic measurements. Michaelis–Menten curves and parameters were estimated using the nonlinear fit (Michaelis–Menten, least squares regression) function in GraphPad Prism v10.3.1.

### Comparison of biokinetic parameters across studies

To enable comparison of N_2_O reduction of bacterial isolates across studies, reported *V*_max_ values were standardized to a common unit of mmol N_2_O g^−1^ cell dry weight h^−1^. Biomass of isolates in this study was estimated from protein content, and we converted it to dry biomass equivalents assuming that total protein accounts for 55% of cellular dry weight [66, 67]. Standardized *V*_max_ values were subsequently used to calculate catalytic efficiency (*V*_max_/*K*_m_) for comparison of N_2_O reduction kinetics among isolates from different ecosystems. Apparent *K*_m_ values were compiled directly from the original studies.

## Results

### Microbial communities in permeable sediments rapidly consume high concentrations of N_2_O

To determine whether N_2_O production occurs *in situ*, we measured N_2_O in pore waters of subtidal sandy sediments at St. Kilda and Werribee beaches located in Port Phillip Bay, a nitrogen-limited marine embayment in Victoria, Australia. Previous studies conducted at these same sites reported active denitrification and measurable N_2_O production potentials in permeable sediments, with maximum rates (*V*_max_) varying from 0.5 to 4.2 nmol g^−1^ h^−1^ [68]. Building on these observations, we expected to detect measurable *in situ* N_2_O in porewater samples. Contrary to this expectation, only trace amounts of N_2_O (~0.0055 μM or 84.6% saturation) were detected in some samples and N_2_O was generally below the limit of detection (0.004 μM dissolved gas concentration) in porewater across most sampling depths and times (Fig. S1, Table S9). The lack of detectable *in situ* N_2_O accumulations is surprising given the denitrification activity previously reported at the same studied sites [47, 68]. Given the analytical detection limits of our measurements was 0.004 μM dissolved N_2_O (or ~50% air–water saturation), we cannot exclude low levels of N_2_O that were present in porewaters, though these findings suggest that N_2_O does not accumulate substantially *in situ.* This may reflect three possibilities (or a combination of): (i) denitrification steps are tightly coupled, producing minimal N_2_O and efficiently reducing it to N_2_; (ii) N_2_O is only sporadically produced when NO_3_^−^ is transported into anoxic zones; or (iii) non-denitrifying bacteria with clade II N_2_O reductases rapidly consume N_2_O produced by other community members.

**Figure 1:**
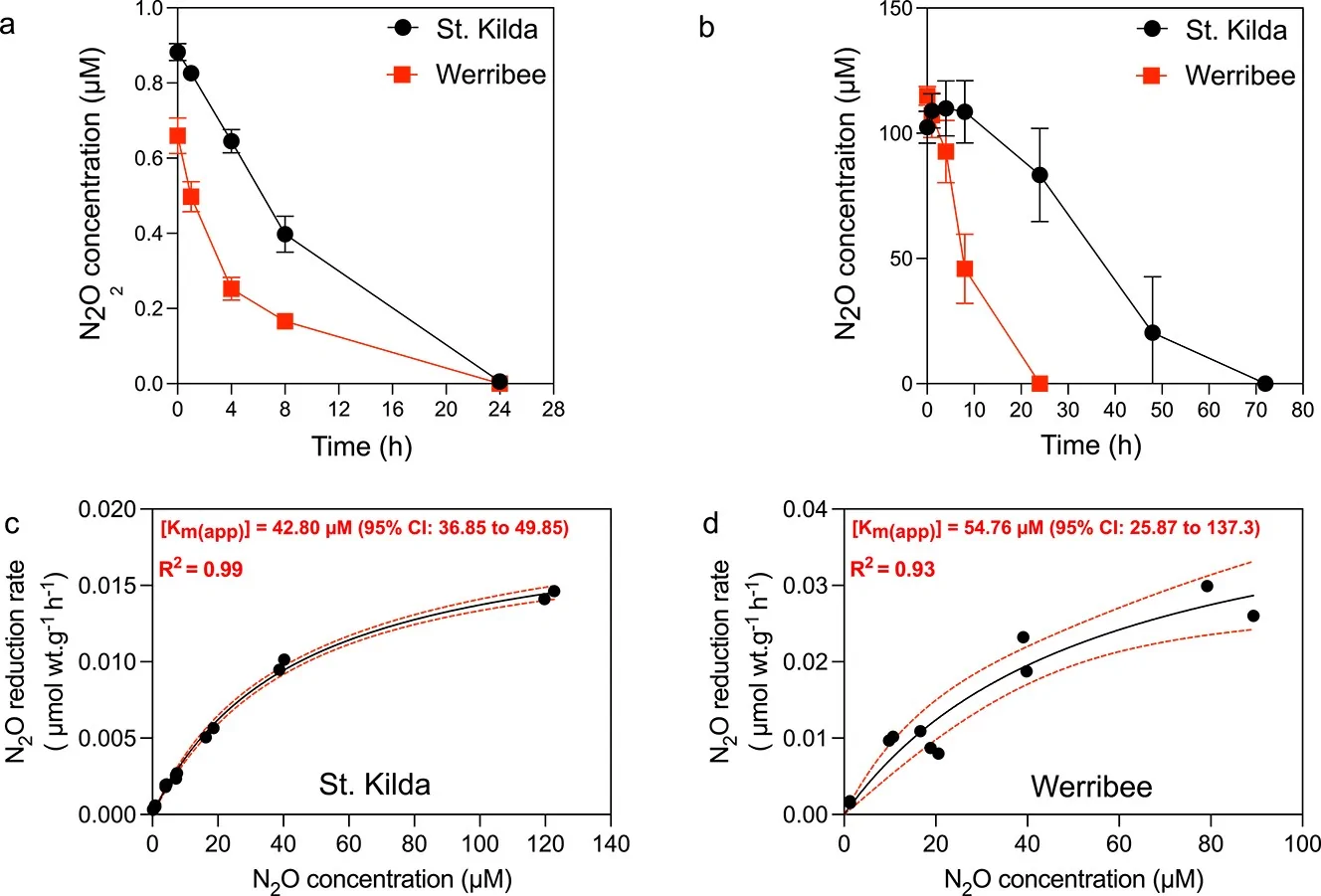
N_2_O consumption by permeable sediments. (a–b) Anoxic slurry incubation of surface sediments (0–5 cm) from St. Kilda (13/05/2024) and Werribee (23/09/2024) with low (~0.8 μM) (a) and high (~100 μM) (b) N_2_O concentrations. (c–d) Kinetics of N_2_O reduction by surface sediments in St. Kilda (13/05/2024 samples) (c) and Werribee (20/08/2025 samples) (d). The curves were fitted, and kinetic parameters were estimated based on a Michaelis–Menten nonlinear regression model.

We performed a series of slurry experiments to determine whether microbial communities within permeable sediments consume N_2_O at a range of concentrations. Under anoxic conditions, microbial communities in the slurries rapidly consumed N_2_O at initial aqueous concentrations of 0.8 and 108 μM, corresponding to headspace mixing ratios of ~10 and 1000ppmv, with the average rates of 0.65 and 49.8 nmol h^−1^ g^−1^ wet sediment, respectively (Fig. 1a). Additionally, Werribee slurries showed significantly higher N_2_O consumption rates (*P* = .015—Student’s *t-*test) than St. Kilda slurries at both concentrations, potentially reflecting long-term enrichment of denitrifying populations in these sediments at the vicinity of a wastewater treatment plant. Kinetic experiments were used to determine whether beach sand microbial communities are adapted to high or low N_2_O concentrations. N_2_O reduction by the sedimentary communities followed Michaelis–Menten-type kinetics (*R*^2^ = 0.99 for St Kilda, 0.93 for Werribee) with apparent half-saturation constants [*K*_m(app)_] of 43 and 55 μM N_2_O, respectively, and maximum reduction rates [*V*_max (app)_] of 19.6 and 46.3 nmol N_2_O h^−1^ g^−1^ wet sediment (Fig. 1b). The [*K*_m(app)_] values observed in this study are unusually high for marine communities, e.g. being orders of magnitude higher than those observed in anoxic estuarine water in Chesapeake Bay, USA (0.65 μM N_2_O) [25] or those in the oxic–anoxic interface from the eastern Pacific oxygen minimum zone (0.33 μM N_2_O) [69]. This suggests that microbial communities within sands are exposed to much higher N_2_O fluxes compared to the water column, presumably due to nutrient enrichment in these ecosystems. Furthermore, the [*V*_max(app)_] for N_2_O reduction in these sediments were ~5- to 90-fold higher than previously reported [*V*_max(app)_] values for denitrification in permeable sediment in Port Phillip Bay (0.5–4.2 nmol g^−1^ h^−1^) [68], implying that the capacity for N_2_O consumption exceeded N_2_O production in these sediments. Altogether, our results suggest that permeable sediments may function as hotspots of N_2_O turnover, where active microbial production and consumption interact to regulate N_2_O dynamics. While permeable sediments could potentially produce elevated N_2_O levels due to nutrient enrichment, rapid microbial N_2_O consumption likely contributes to limiting its accumulation and emissions, consistent with the below saturation N_2_O concentrations observed in porewater samples. Despite this, we also cannot exclude the possibility that tightly coupled denitrification or physical transport process also contribute to the low porewater N_2_O concentrations observed in this study.

### Permeable sediments host diverse N_2_O producers and consumers

**Figure 2:**
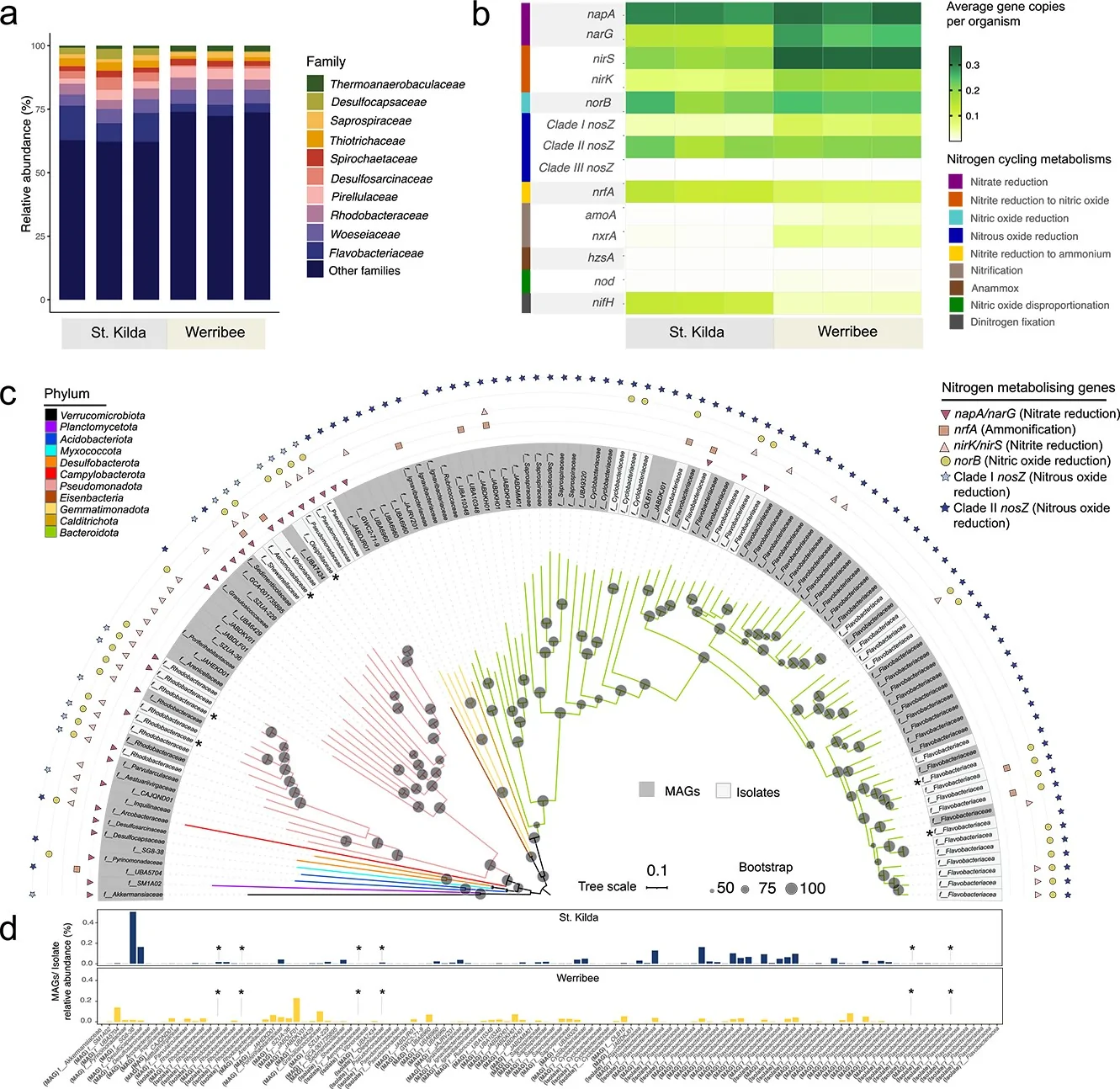
Nitrogen-cycling metabolic capacity of microbial communities in permeable sediments. (a) Family-level microbial community composition of surface sediments at St. Kilda and Werribee beaches reconstructed from 16S rRNA genes by PhyloFlash. (b) Relative abundance of nitrogen metabolizing genes in St. Kilda and Werribee beaches based on gene-centric metagenomic analysis (c) Maximum-likelihood phylogenomic tree showing nitrogen-cycling metabolic gene inventories of representative isolates and bacterial MAGs generated from this study and acquired from [47, 48]. The tree was built with 1000 ultrafast bootstrap replicates using the LG + F + I + G4 model via IQ-TREE. Symbols indicate the presence of key metabolic genes for denitrification. (d) Relative abundance of representative isolates and MAGs at two studied sites estimated by mapping short reads to genomes using CoverM. Specific isolates were selected for further analysis, including culture-based activity and kinetic measurements (asterisks).

We profiled microbial community composition of permeable sediment by sequencing metagenomes from each site and taxonomically assigning ribosomal small subunit genes using PhyloFlash (Fig. 2a, Table S2). Consistent with previous reports at the same studied sites [47, 48], *Flavobacteriaceae* was the most abundant family in the sediments (~10% relative abundance), while *Woeseiaceae* and *Rhodobacteriacae* were also abundant across the sediments (Fig. 2a, Table S2). We then used genome-resolved metagenomics to better understand the microbial and enzymatic mediators of N_2_O cycling in permeable sediments. We sequenced and performed *de novo* co-assembly and binning of metagenomes from the Werribee and St. Kilda beaches, yielding 53 medium-quality and 17 high-quality MAGs. We additionally reanalysed 179 MAGs we previously obtained from St. Kilda permeable sediments [47, 48] (Table S4). Overall, the resultant 249 MAGs span 19 phyla and 74 orders including the three aforementioned highly abundant families (Fig. S2, Table S4). We then annotated the short reads, derived MAGs, and unbinned contigs to determine the functional capacities and diversity of the permeable sediment microbiome.

In line with previous studies [47, 48], microbial communities within permeable sediments have high capacity for denitrification. Specifically, many community members are predicted to mediate NO_3_^−^ reduction via Nar and Nap (St. Kilda: 44%; Werribee: 58%), NO_2_^−^ reduction via NirS and NirK (St. Kilda: 27%; Werribee: 54%), and nitric oxide (NO) reduction to N_2_O via Nor (St. Kilda: 22%; Werribee: 24%) (Fig. 2b; Table S3). Metabolic reconstruction showed that 76 MAGs (30%) encode partial denitrification pathways, i.e. MAGs missing one or more denitrifying reductases (Table S4). To minimize artefacts of missing genes due to fragmented genomes, we performed more focused analyses of 64 high-completeness (>90%) and low-contamination (<5%) MAGs to more reliably infer partial denitrification pathways. Among these, 16 MAGs were found to possess one or more denitrifying reductases but lack the N_2_O reductases (Fig. 2C). MAGs associated with *Arenicellaceae* (*Gammaproteobacteria*), *JABDKV01* (*Gammaproteobacteria*), and *Pyrinomonadaceae* (*Acidobacteriota*) families encoded Nor for N_2_O production, suggesting that these microorganisms are N_2_O producers within the sand communities. Compared to denitrification, fewer community members mediate nitrification via Amo and Nxr (St. Kilda: 0.4%; Werribee: 8%) or dissimilatory nitrate reduction to ammonium (DNRA via Nrf—St. Kilda: 13%; Werribee: 9%) (Fig. 2b; Table S3).

Consistent with our activity measurements, analysis of metagenomic short reads showed that many bacteria encode genes to reduce N_2_O via NosZ (St. Kilda: 23.3%; Werribee: 29.1%; Fig. 2b). In detail, bacteria encoding the NosZII are more abundant than the NosZI (19.6% and 20.2% vs 3.5% and 8.7% of St. Kilda and Werribee communities, respectively) while only a minor fraction of the community (St. Kilda: 0.01%; Werribee: 0.8%) encoded the NosZIII (Fig. 2b). Consistently, at the MAG level, only five MAGs associated with *Pseudomonadota* (*n* = 4) and *Verrucomicrobiota* (*n* = 1) encode NosZI, whereas 61 MAGs spanning 8 phyla encode the NosZII (Fig. 2b). Of NosZII-containing MAGs, 49 (80%) were Bacteroidota and 57% of these were *Flavobacteriaceae* family. Screening of denitrification marker genes (i.e. *napA*, *narG*, *nirS*, *nirK*, and *norB*) showed that 11 of 58 clade II *nosZ*-containing MAGs (19%) co-encoded additional denitrification genes. Among these, five MAGs encoded *nosZ* together with either *nirS* or *nirK* only, four MAGs encoded *nosZ* together with either *napA* or *narG* only, and one MAG (classified as *Arcobacter* sp.001655195) encoded the complete denitrification pathway (*nar*, *nir*, *nor*, and clade II *nosZ*). In contrast, 47 MAGs (81%) lacked upstream denitrification genes. Notably, 12 of these 47 MAGs exhibited high completeness (>90%) and low contamination (<5%), supporting that the absence of upstream denitrification genes in these genomes is unlikely to result from incomplete assembly. However, absence of upstream denitrification genes in the remaining medium-quality MAGs should be interpreted more cautiously. Consistent with these genome-resolved patterns, analysis of clade II *nosZ* associated reads based on best DIAMOND hits showed that ~36% were affiliated with *Flavobacteriaceae*, indicating that this family represents a substantial component of the clade II N_2_O reducing community in the sediments (Table S3). Together, these results suggest that a substantial subset of clade II *nosZ*-containing organisms may have the genetic potential for N_2_O reduction without a complete canonical denitrification pathway, thereby functioning primarily as N_2_O consumers rather than producers.

**Figure 3:**
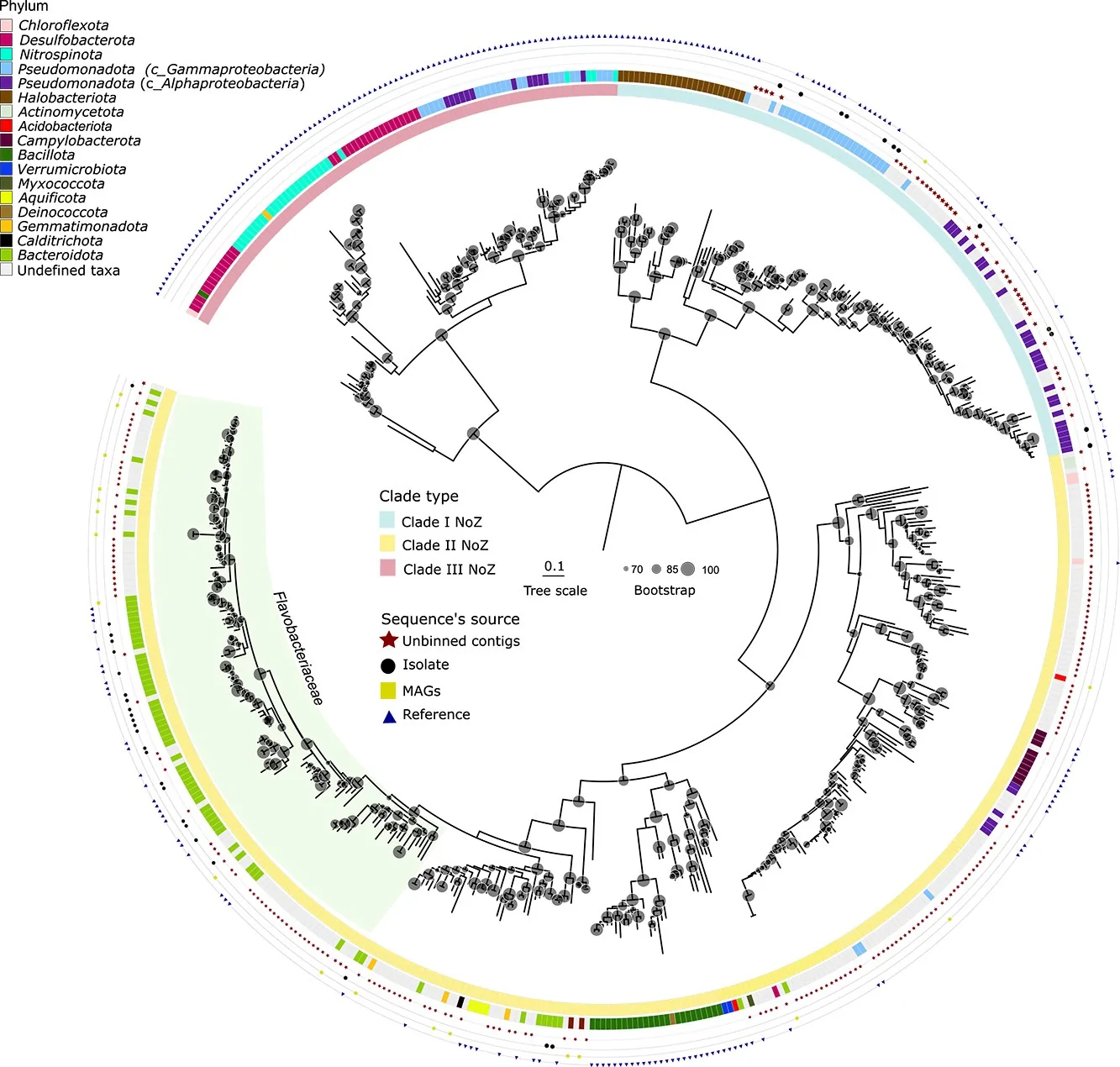
Diversity of NosZ in permeable sediments. The maximum-likelihood phylogenetic tree of NosZ amino acid sequences was generated using LG + I + G4 substitution model (via IQ-TREE) and was midpoint rooted. Symbol indicates the source of NosZ amino acid sequences, including those derived from MAGs (*n* = 24), isolate genomes (*n* = 36), unbinned contigs (*n* = 237), and 250 reference NosZ sequences. The inner ring indicates the NosZ clade while the outer ring shows taxonomic assignments.

To gain further insight into the phylogenetic diversity of the N_2_O reducers, we constructed a phylogenetic tree of NosZ including 547 amino sequences retrieved from 36 isolate genomes, 24 MAGs, 237 unbinned contigs, and 250 reference sequences (Fig. 3). Consistent with previous findings [27, 29, 70, 71], our phylogenetic tree revealed that the NosZI is predominantly restricted to the *Pseudomonadota*, whereas the NosZII showed broader taxonomic distribution across several phyla (Fig. 3). Most NosZII sequences affiliate with *Bacteroidota*, specifically the family *Flavobacteriaceae* (Fig. 3). NosZII sequences were also affiliated with other phyla, including *Gemmatimonadota*, *Acidobacteriota*, and *Chloroflexota*. While the NosZIII was reported to be associated with diverse taxa [26], this gene was not detected in the isolate genomes or assembled contigs in our dataset, suggesting low prevalence of clade III N_2_O reducers in the studied sandy sediments.

### An isolate collection of marine microbes uncovers novel mediators of N_2_O cycling

To complement the metagenomic insights into the potential mediators of N_2_O cycling, we conducted an isolation campaign to obtain high-quality genomes, confirm metabolic activities, and gain ecophysiological insights into key N_2_O-cycling microorganisms. To the best of our knowledge, this cultivation effort generated the largest collection of permeable sediment isolates (*n* = 95) to date. These spanned four main phyla, namely *Bacillota* (*n* = 6), *Actinobacteriota* (*n* = 4), *Bacteroidota* (*n* = 36), *Alphaproteobacteria* (*n* = 29), and *Gammaproteobacteria* (*n* = 20). These isolates capture a considerable fraction of the community’s phylogenetic diversity observed from metagenome-resolved data, including several lineages clustering with MAG-derived clade II *nosZ-*containing populations from the sediments (Fig. 2c, Fig. S2). Based on GTDB, our isolate collection includes two novel bacterial genera and 57 novel bacterial species (Table S5). To assess if these cultivated strains correspond to environmentally occurring populations, metagenomic reads were mapped to both isolate genomes and MAGs using CoverM to estimate their relative abundance across sediment metagenomes (Fig. 2d, Table S5). MAG-derived populations were generally more abundant than isolate genomes; however, the isolates still captured several moderately abundant lineages, including *Flavobacteriaceae* genera *Murricola* (~0.06% relative abundance) and *Maribacter* (~0.03% relative abundance). Thus, while not direct proxies for dominant populations, these isolates provide experimentally tractable representatives of environmentally relevant N_2_O reducing lineages.

**Figure 4:**
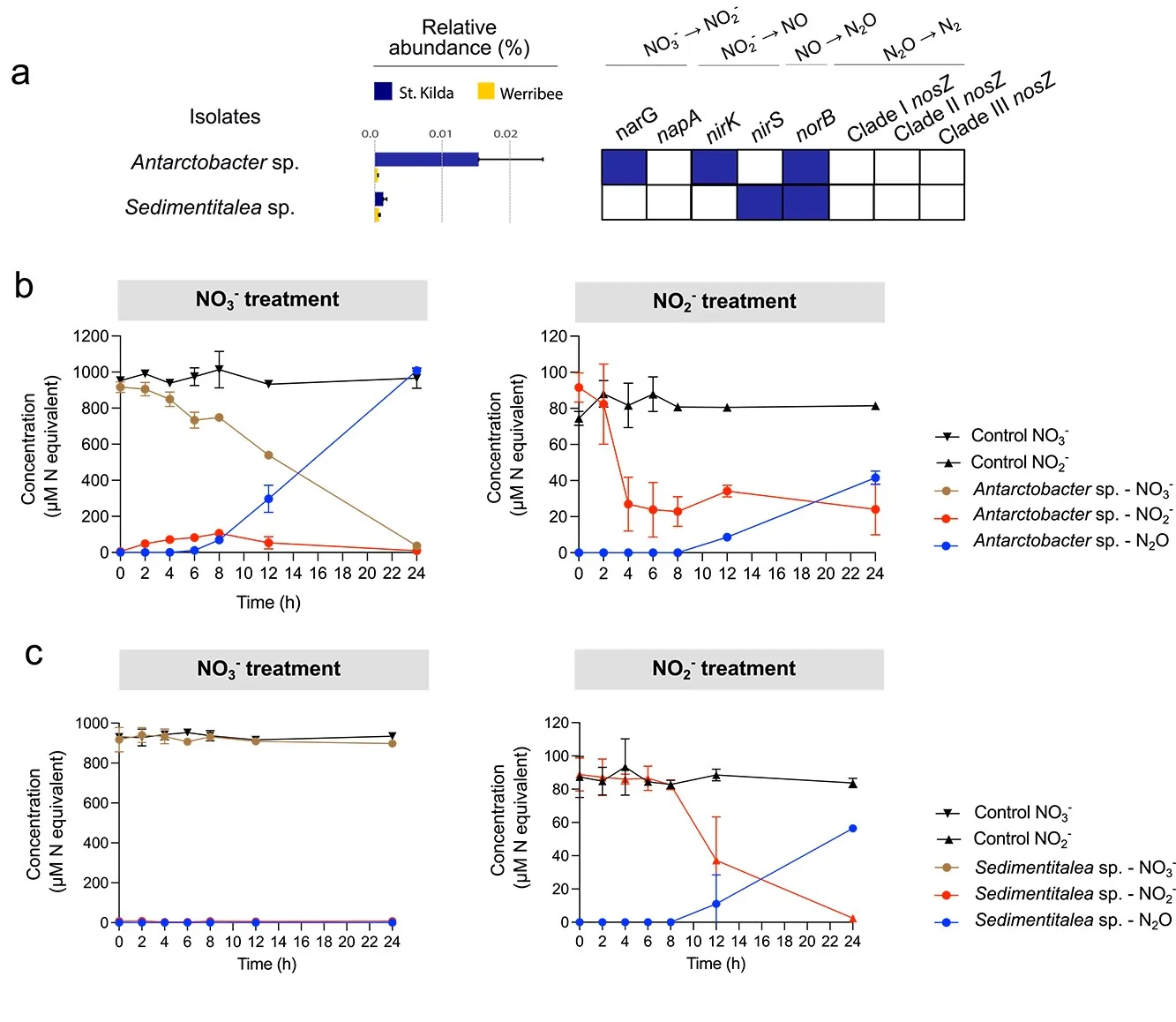
Partial denitrification genotype and phenotype of selected bacterial isolates. (a) Relative abundance (% of total reads per sample) and denitrification gene content of two isolates. Relative abundance across sediment metagenomes is shown for two sites (St. Kilda and Werribee) and was estimated by mapping metagenomic reads to isolate genomes using CoverM. Presence and absence of denitrification genes are indicated for each isolate. (b) Partial denitrification phenotypes in *Antarctobacter* sp. (c) Partial denitrification phenotypes in *Sedimentitalea* sp. Both isolates were grown on Difco 2216 Marine Broth with either 1 mM NaNO_3_ for NO_3_^−^ treatment or 0.1 mM NaNO_2_ for NO_2_^−^ treatment under anoxic conditions. Data are presented as mean ± SD, *n* = 3 biologically independent samples, with media-only vials (*n* = 2) as negative controls.

Among these isolates, five *Alphaproteobacteria* (*Rhodobacteraceae)* and seven *Gammaproteobacteria* encode a complete set of denitrifying genes, including clade I *nosZ*, indicating capacity to completely reduce NO_3_^−^ to N_2_. Another 12 proteobacterial isolates encode denitrification genes except *nosZ*, suggesting that they produce N_2_O through partial denitrification. The partial denitrification phenotype was experimentally validated in two isolates, an *Antarctobacter* sp. and *Sedimentitalea* sp. (*Rhodobacteraceae*, Alphaproteobacteria), which were detected at low relative abundance (<0.02%) in sediment metagenomes (Fig. 4, Table S5). The *Antarctobacter* sp. has the genomic capacity to mediate stepwise NO_3_^−^ reduction to N_2_O (NO_3_^−^ → NO_2_^−^ → NO → N_2_O) via Nar, NirK, and Nor, while *Sedimentitalea* sp. is predicted to only mediate NO_2_^−^ reduction to N_2_O (NO_2_^−^ → NO → N_2_O) via NirS and Nor (Fig. 4b, c, Table S8). Consistently, the *Antarctobacter* sp. isolate completely reduced 917 ± 30 μM NO_3_^−^ to N_2_O after 24 h of incubation with the expected 1:1 stoichiometry, after transiently producing small quantities of NO_2_^−^. As expected, the strain also produced N_2_O when supplemented with NO_2_^−^; however, this was a less efficient process and only ~62% of the initial NO_2_^−^ was reduced to N_2_O after 24 h of incubation, suggesting delayed NO reduction (Fig. 4b, Table S8). Also consistent with the genomic predictions, the *Sedimentitalea* sp. isolate could not consume NO_3_^−^ but did convert NO_2_^−^ to N_2_O after an initial lag (Fig. 4c, Table S8). Given both isolates lacked the N_2_O reductases, they were unable to perform the last step of denitrification and thereby emitted N_2_O as their primary end product.

By contrast, most isolates showed widespread capacity for N_2_O reduction. Clade I *nosZ* genes were identified in the 12 Proteobacteria capable of complete denitrification. In contrast, clade II *nosZ* genes were identified in 20 *Flavobacteriaceae* and 3 *Cyclobacteriaceae* isolates (Bacteroidota). Among these, 4 encoded the clade II *nosZ* without any denitrifying genes, while 16 co-encoded *nosZ* with *nor* for NO reduction to N_2_O. Notably, some *Flavobacteria*ceae isolates (e.g. *Lutibacter* sp., *Algibacter* sp., and *Winogradskyella* sp.) also encoded *nrf* for DNRA, whereas others (e.g. *Arenibacter algicola* or *Xanthomarina* sp.) also encoded the *nirK* genes for NO_2_^−^ reduction. Whereas metagenomic predictions indicate that the most dominant *Flavobacteriaceae* lineages within permeable sediments are exclusive N_2_O reducers, results from microbial isolates suggest that *Flavobacteriaceae* members can variably mediate NO_2_^−^ reduction, DNRA along with N_2_O reduction. This discrepancy likely reflects biases introduced during cultivation, where enrichment and medium conditions may favour organisms capable of utilizing a broader range of nitrogen species as electron acceptors.

Further genomic analyses on these isolates revealed their broader metabolic breadth (Table S5). Most of them encode multiple terminal oxidases for aerobic respiration while some (e.g. *Lutibacter* sp. or *Oceanimonas* sp.) also encode group 3b or 3d [NiFe]-hydrogenases, which contribute to fermentative H_2_ production within these sediments as previously reported [14]. This suggests that N_2_O-cycling microorganisms can be extremely versatile switching between aerobic respiration, anaerobic respiration, and fermentation depending on availability of electron acceptors. Additionally, many isolates encode sulphide-quinone oxidoreductase (Sqr) for sulphide oxidation, while others encoded carbon monoxide dehydrogenase for trace gas oxidation or rhodopsin for phototrophy, suggesting that permeable sediment microbes can variably use organic, inorganic, and solar energy sources. Altogether, these culture-based insights support our previous genome-resolved metagenomic predictions [47, 48] that permeable sediment microorganisms are highly metabolically flexible, possibly as a response to frequent environmental fluctuations.

### Clade II *nosZ*-encoding bacteria from permeable sediments show low affinity towards N_2_O

**Figure 5:**
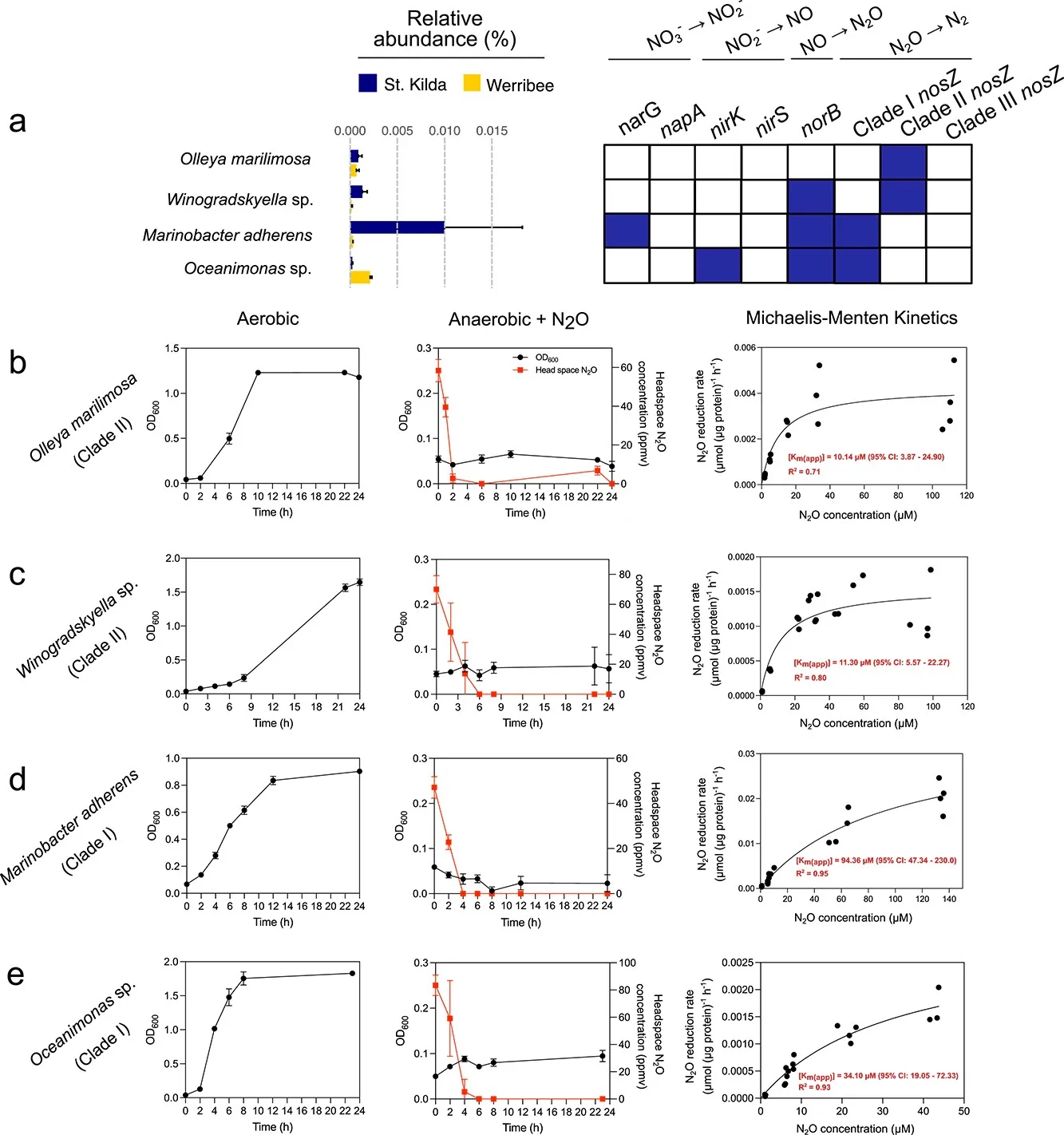
N_2_O reduction genotype and phenotype of clade I and II N_2_O reducing isolates. (a) Relative abundance (% of total reads per sample) and denitrification gene content of four isolates. Relative abundance across sediment metagenomes is shown for two sites (St. Kilda and Werribee) and was estimated by mapping metagenomic reads to isolate genomes using CoverM. Presence and absence of denitrification genes are indicated for each isolate. (b–d) Aerobic growth and N_2_O reducing activity of *O. marilimosa* and *Winogradskyella* sp. (clade II *nosZ* isolates) and *M. adherens* and *Oceanimonas* sp. (clade I *nosZ* isolates), respectively. Isolates were grown on Difco 2216 Marine Broth aerobically or anaerobically with N_2_O as the sole terminal electron acceptor. Growth was determined by optical density measurements at 600 nm, followed by measurements of N_2_O concentration in the headspaces of the culture vials. Kinetics of N_2_O reduction of four isolates were calculated based on a Michaelis–Menten nonlinear regression model. All experiments were performed in triplicate. Data are presented as mean ± standard deviation (SD).

To further determine the physiological characteristics of N_2_O reducers in permeable sediments, we investigated N_2_O consumption by four marine bacterial isolates capable of aerobic organoheterotrophic growth, which were detected at low relative abundance (<0.02%) in sediment metagenomes (Fig. 5a). Two strains, *O. marilimosa* and *Winogradskyella* sp. (both *Flavobacteriaceae*), carry the clade II *nosZ* gene but lack key denitrification genes (i.e. *nirS* or *nirK*). In contrast, *Marinobacter adherens* (*Oleiphilaceae*) and *Oceanimonas* sp. (*Aeromonadaceae*) carry denitrification genes and clade I *nosZ*. These four isolates grew aerobically in Difco 2216 Marine Broth using yeast extract and peptone as the carbon and energy sources (Fig. 5a, d, g, and  j, Table S7) and quickly reduced all headspace N_2_O under anoxic conditions (Fig. 5b, e, h, and k, Table S7). Whole-cell kinetic measurements revealed that N_2_O reduction followed Michaelis–Menten-type kinetics in all isolates (Fig. 5c, f, i, and l). The mean apparent half-saturation constants [*K*_m(app)_] of clade I *nosZ* strains *M. adherens* and *Oceanimonas* sp. were 94.4 μM (95% CI: 47–230) and 34.1 μM (95% CI: 19–72). In contrast, [*K*_m(app)_] values of clade II *nosZ* flavobacterial isolates *O. marilimosa* and *Winogradskyella* sp. were considerably lower at 10.1 μM (95% CI: 3.9–25) and 11.3 μM (95% CI: 5.6–22.3), respectively.

**Figure 6:**
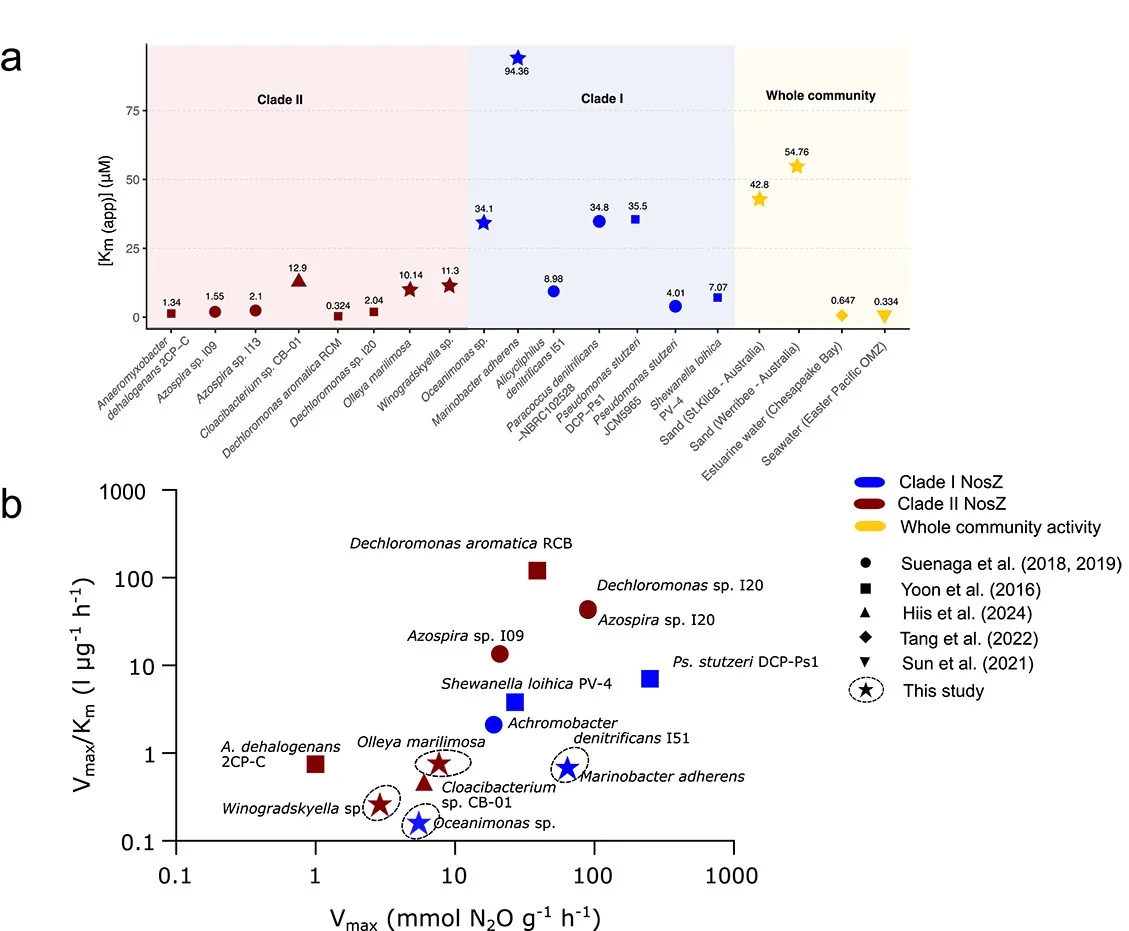
Comparison of N_2_O reduction biokinetics between different N_2_O-reducing microorganisms. (a) Affinities for N_2_O reported for N_2_O-reducing isolates and whole communities from this and previous studies [25, 69, 72–75]. (b) N_2_O sink strength of various microorganisms from different ecosystems, determined by plotting the ‘catalytic efficiency’ (*V*_max_/*K*_m_) against the maximum reduction rates (*V*_max_). To enable consistent comparison, we converted all *V*_max_ values to mmol N_2_O g^−1^ cell dry weight h^−1^, assuming the total protein amounts account for 55% of the total dry biomass [66, 67]. All values, except that reported in [78] (23°C), were measured at 30°C.

We subsequently compared the N_2_O sink strength of our permeable sediment isolates with previously characterized N_2_O-reducing bacteria from other ecosystems (Fig. 6). Clade II *nosZ* flavobacterial isolates in this study had much higher [*K*_m(app)_] values than most clade II *nosZ* microorganisms reported to date such as *Anaeromyxobacter dehalogens* (1.34 μM) [72] or *Dechloromonas aromatica* RCM (0.32 μM) [73] (Fig. 6a), indicating a relatively low affinity for N_2_O. In addition to substrate affinity, we also compared ‘catalytic efficiencies’ (*V*_max_/*K*_m_) among the isolates to understand their ability to reduce N_2_O under low N_2_O concentrations (Fig. 6b). Because biomass specific *V*_max_ could not be determined for whole-sediment incubations, catalytic efficiencies derived from sediment measurements were not directly compared with isolate values. Therefore, comparisons between isolates and sediments incubations focus on apparent *K*_m_ values, which can be interpreted independently of biomass normalization (Fig. 6a). While the maximum reduction rates (*V*_max_) of our isolates (except the clade I NosZ-containing *M. adherens*) were comparable to the average of the other bacteria, their ‘catalytic efficiencies’ (*V*_max_/*K*_m_) was low due to their low apparent affinity towards N_2_O (Fig. 6b, Table S6). These results indicate that under the experimental conditions tested here, our isolates have relatively weak N_2_O sink strength under both high and low substrate conditions compared with previously characterized N_2_O reducing bacteria.

## Discussion

Through integrative approaches, we demonstrate that N_2_O cycling in permeable sediment is mediated by a delicate balance between microbial production and consumption. Consistent with previous studies [34, 47, 48], our results show that permeable sediments host diverse microorganisms with partial denitrification pathways (i.e. lacking one or more genes required for complete denitrification). Some of the most abundant members of the community (e.g. *Rhodobacteraceae* members) possessed either nitrite reductase and/or nitric oxide reductase, but lacked NosZ, thus producing N_2_O as the terminal product of denitrification and contributing to N_2_O emissions. Furthermore, we experimentally confirmed this partial genotype in two *Rhodobacteraceae* isolates (i.e. *Antarctobacter* sp. and *Sedimentitalea* sp.), which produced N_2_O from either NO_3_^−^ or NO_2_^−^ without further reducing it to N_2_, validating metagenomic and culture-based genomic predictions. At the ecosystem level, denitrification is recognized as a collective effort performed by different community members and partial denitrification can be more common across diverse terrestrial and aquatic environments [21, 76, 77]. Partitioning of denitrification steps among community members could alleviate competition between enzymes acting on the same substrates and enhance community flexibility and resilience under environmental disturbances [76]. Furthermore, specializing in a certain step allows partial denitrifiers to minimize resource allocation to denitrification, enabling broader metabolic flexibility to adapt to complex, dynamic, and oligotrophic ecosystems [77]. Given that permeable sediments are highly dynamic with strong spatiotemporal variation in electron donor and acceptor availability, partial denitrification is more prevalent than complete denitrification in these systems. Although we did not directly measure N_2_O production rates in this study, the high [*K*_m(app)_] values for N_2_O reduction of St. Kilda and Werribee sediments suggest that the N_2_O reducing community in these sites is adapted to high N_2_O concentrations potentially generated by these partial denitrifiers.

Despite substantial capacity for N_2_O production, we provide biogeochemical, metagenomic, and culture-based evidence that most N_2_O is consumed by the sediment community before being emitted into the atmosphere. A diverse consortium of N_2_O-reducing microorganisms carrying the clade I and II *nosZ* gene were detected, which efficiently reduce N_2_O to N_2_ before being released into the atmosphere. These microorganisms span several phyla including *Bacteroidota*, *Pseudomonadota*, *Campylobacterota*, and *Gemmatimonadota*, with metabolically versatile members of the *Flavobacteriaceae* (*Bacteroidota*) carrying clade II *nosZ* genes particularly dominant. Our results indicate that most flavobacterial isolate genomes and MAGs lack canonical genes required for dissimilatory denitrification, suggesting that these bacteria are unlikely to produce N_2_O via the conventional denitrification pathway. Instead, by expressing the clade II *nosZ* gene, they function as N_2_O respirers that scavenge N_2_O generated by other microorganisms. Moreover, their N_2_O-reducing capacity, together with the ability to use diverse energy sources, electron donors, and electron acceptors, reflects their metabolic flexibility that allows them to thrive under frequent oxic–anoxic shifts of these sediments. By combining metagenome-resolved and isolate genomic analyses, we can gain insight into the physiology potential of these bacteria to persist under the highly dynamic conditions of permeable sediments that are difficult to fully reproduce in laboratory incubations. In this study, clade III *nosZ* was scarcely detected in the top sediment layers. Previous work has suggested that clade III *nosZ*-carrying bacteria, such as members of *Desulfobacterota* or *Nitrospinota* phyla, are more likely to inhabit anoxic environments [26], whereas the highly dynamic redox conditions of permeable sediments likely limit their occurrence.

In rippled permeable sediments where solutes such as NO_3_^−^ and N_2_O are continually exchanged via advective porewaters [9, 78], N_2_O-reducing microorganisms might be expected to rapidly scavenge N_2_O with great sink strength. This hypothesis was based on previous reports suggesting that clade II *nosZ*-containing microorganisms possess high affinity for N_2_O, together with our observation of few detectable *in situ* N_2_O concentrations, which led us to hypothesize that representative clade II N_2_O reducers isolated from permeable sediments would exhibit highly efficient N_2_O reduction. However, contrary to our expectation, our physiological work showed that bacterial isolates from permeable sediments exhibited relatively weak apparent N_2_O-reducing capacity under the experimental conditions tested, as reflected in both average maximum reduction rates (*V*_max_) and low catalytic efficiency (*V*_max_/*K*_m_) compared with other known N_2_O-reducing bacteria. This unexpected finding raises questions about the mechanisms regulating N_2_O cycling within these sediments.

Based on previous hydrodynamic and reactive transport studies of permeable sediments [38, 79–81], one possible explanation lies in the coupling between microbial activity and porewater transport dynamics. The majority of denitrification in permeable sediments is driven by NO_3_^−^ fed from overlying water [80, 82] while advective flow and transport dynamics can spatially separate oxic and anoxic zones [80]. Under calm conditions without physical disturbances such as intense waves, once NO_3_^−^ enters the reduced reaction zone and is denitrified to N_2_O, porewater residence time within ripple structures, typically on the order of hours to days depending on flow conditions [81], may allow N_2_O reducers to fully reduce N_2_O to N_2_. This is consistent with our experimental observation that N_2_O at elevated concentrations (~10 ppmv) was completely consumed within 24 h, indicating that microbial reduction can occur on similar timescales. Given that these concentrations likely exceed *in situ* N_2_O levels, consumption in natural sediments is expected to occur on comparable or shorter timescales. This hypothesis is further supported by previous models showing that denitrification in permeable sediments is strongly structured by advective transport and can proceed efficiently within subsurface zones, with limited release of intermediate products [79, 80]. Consistently, previous model simulations showed that under calm wave conditions which are predominant at these studied sites, N_2_O efflux from sediments is minimal and associated with high Damköhler numbers, reflecting fast reaction rates compared with transport processes [38]. Conversely, under more hydrodynamically active conditions, more oxygen is introduced into the sediment, limiting the opportunities for denitrification and N_2_O production to take place. It is only under specific intermediate conditions of sediment flushing that significant amounts of N_2_O escapes the sediment, making it a rare occurrence (<3% of the time) [38]. These observations suggest a potential link between microbial physiology and sediment transport dynamics that may help explain why dominant clade II N_2_O reducers from permeable sediments exhibited relatively low apparent affinity for N_2_O under the experimental conditions tested. However, as N_2_O residence times and porewater transport dynamics were not directly measured, and kinetic parameters may vary with experimental conditions, this interpretation should be considered a hypothesis rather than a demonstrated ecological mechanism. Future work integrating microbial physiology with *in situ* transport processes will be needed to resolve how hydrodynamics and microbial metabolism regulate N_2_O cycling in permeable sediments.

Altogether, our findings suggest a potential cross-feeding interaction between N_2_O producers and non-denitrifying N_2_O reducers that may contribute to buffering the emissions of this potent greenhouse gas in eutrophic permeable sediments. Our results further indicate that the capacity for N_2_O removal by clade II N_2_O-reducing bacteria may be linked to the physical characteristics of these sediments rather than solely to their physiological characteristics. These results suggest that sediment transport dynamics can shape greenhouse gas cycling and influence ecophysiological strategies of resident microbial communities. Future work should investigate how these microorganisms respond and mediate N_2_O emissions under variable hydrodynamic regimes.

## Supplementary Material

Web_Material_wrag186

## Data Availability

All sequences generated from this work were deposited to the NCBI Sequence Read Archive under the BioProject accession numbers PRJNA1347951 for metagenomes, PRJNA1357641 for metagenome-assembled genomes, and PRJNA1348900 for isolate whole genomes. Bacterial and archaeal MAGs generated from the same sites in previous studies [47, 48] were downloaded from NCBI Sequence Read Archive with BioProject accession numbers PRJNA623061 and PRJNA609151.
