# Supplementary material for: Flavobacteria consume nitrous oxide produced by partial denitrifiers in coastal sediments
Source: ISME J. 2026 Jul 14;20(1):wrag186. doi: 10.1093/ismejo/wrag186 (PMC13502197; doi:10.1093/ismejo/wrag186)
Supplement: Supplementary file 1 [file web_material_wrag186.zip › FigS1.pdf]

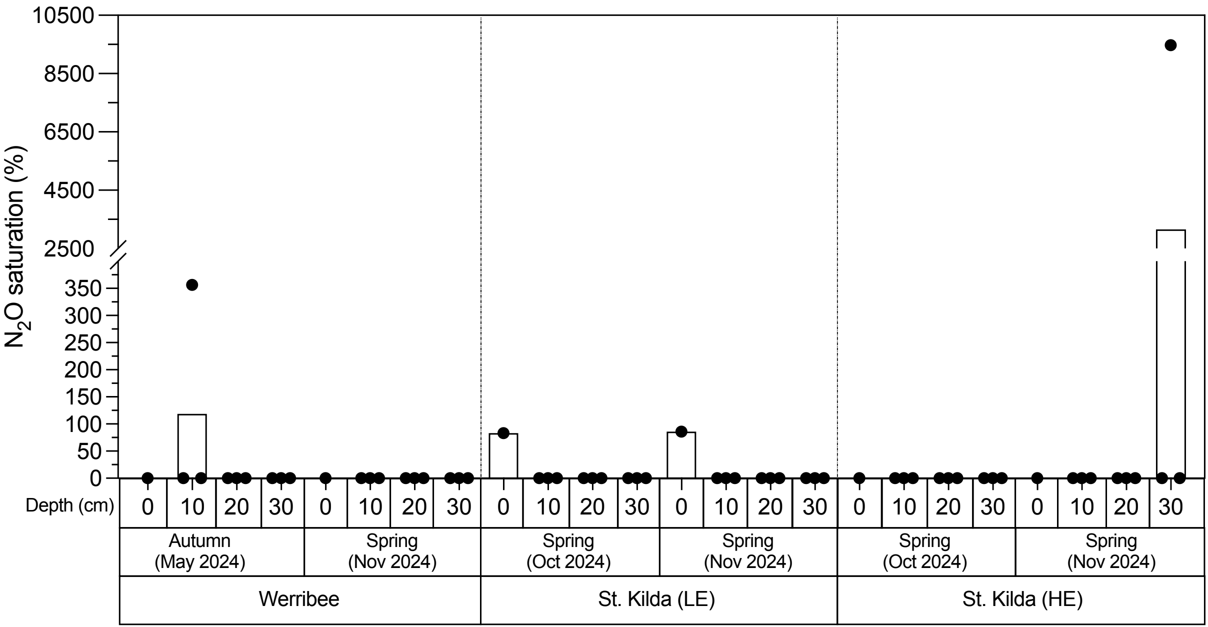

N2O saturation (%)
10500
8500
6500
4500
2500
350
300
250
200
150
100
50
0
Depth (cm)
0
10
20
30
Autumn (May 2024)
Spring (Nov 2024)
Spring (Oct 2024)
Spring (Nov 2024)
Spring (Oct 2024)
Spring (Nov 2024)
Werribee
St. Kilda (LE)
St. Kilda (HE)
