# Supplementary material for: Flavobacteria consume nitrous oxide produced by partial denitrifiers in coastal sediments
Source: ISME J. 2026 Jul 14;20(1):wrag186. doi: 10.1093/ismejo/wrag186 (PMC13502197; doi:10.1093/ismejo/wrag186)
Supplement: Supplementary file 1 [file web_material_wrag186.zip › Figure S2.pdf]

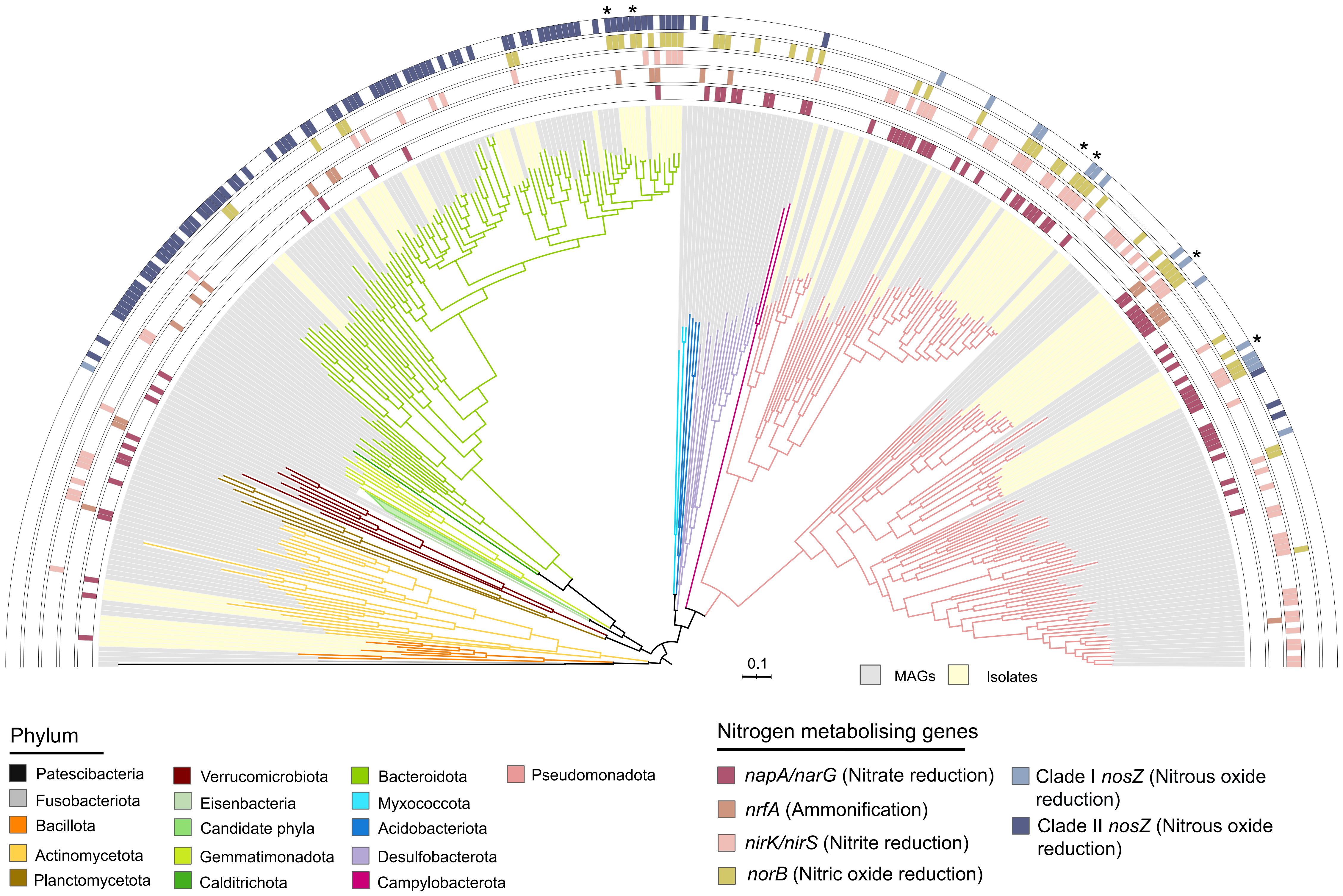

*
0.1
MAGs
Isolates
Phylum
Patescibacteria
Fusobacteriota
Bacillota
Actinomycetota
Planctomycetota
Verrucomicrobiota
Eisenbacteria
Candidate phyla
Gemmatimonadota
Calditrichota
Bacteroidota
Myxococcota
Acidobacteriota
Desulfobacterota
Campylobacterota
Pseudomonadota
Nitrogen metabolising genes
napA/narG (Nitrate reduction)
nrfA (Ammonification)
nirK/nirS (Nitrite reduction)
norB (Nitric oxide reduction)
Clade I nosZ (Nitrous oxide reduction)
Clade II nosZ (Nitrous oxide reduction)
