# Supplementary material for: Flavobacteria consume nitrous oxide produced by partial denitrifiers in coastal sediments
Source: ISME J. 2026 Jul 14;20(1):wrag186. doi: 10.1093/ismejo/wrag186 (PMC13502197; doi:10.1093/ismejo/wrag186)
Supplement: Supplementary file 1 [file web_material_wrag186.zip › Supplementary Information_Revised.docx]

**Supplementary Information**

Flavobacteria consume nitrous oxide produced by partial denitrifiers in coastal sediments

**Supplementary Tables**

**Table S1 (xlsx)** Composition of different media used for isolation of bacteria from permeable sediments in St. Kilda Beach and Werribee.

**Table S2 (xlsx)**. PhyloFlash OUT table and Family level relative abundance

**Table S3 (xlsx).** Sequencing statistics and abundance of metabolic marker genes in the short read metagenomic data. This includes the calculated average copies per organism for each marker gene, average copies per organism for nitrogen metabolising genes, a list of short read hits and their corresponding match in the database and a list of short read hits corresponding to clade II *nosZ* sequences and their associated taxonomy.

**Table S4 (xlsx).** Summary and annotation of dereplicated metagenome-assembled-genomes (MAGs) obtained from this study and previous [1,2] studies. This includes taxonomic information for each MAG, relative abundance of each MAG per sample calculated by CoverM, a summary of metabolic marker genes identified in each MAG, a list of clade II *nosZ* containing MAGs, a list of high completeness and low contamination MAGs and a full list of metabolic marker genes identified in the MAGs with alignment information and protein sequences, and sequences of metabolic genes in unbinned contigs derived from homology-based searches.

**Table S5 (xlsx).** Summary of bacterial isolates. This includes taxonomic information for each isolate, relative abundance of isolate genomes in the sediment metagenomes estimated via read mapping, and a summary of metabolic marker genes identified in each isolate genome.

**Table S6.** Comparison of biokinetic parameters for marine permeable sediment isolates and other N_2_O-reducing bacteria.

**Table S7 (xlsx).** Measurements of N_2_O concentrations and kinetic analyses. This includes measurement of N_2_O concentrations in slurries and four bacterial isolates.

**Table S8 (xlsx).** Measurements of NO_x_ and N_2_O in two *Rhodobacteraceae* isolates (i.e. *Antarctobacter* sp. and *Sedimentitalea* sp.) with partial denitrification pathways.

**Table S9 (xlsx).** *In situ* N_2_O concentrations in porewaters of St. Kilda and Werribee permeable sediments.

**Supplementary Figures**

**Figure S1. *In situ*** **N_2_O concentrations in permeable sediments.** Pore-water (0, 10, 20, and 30 cm) concentrations of N_2_O collected at Werribee (24/05/2024 and 05/11/2024) and St. Kilda beaches at low energy site (LE) and high energy site (HE) (15/05/2024, 01/10/2024 and 18/11/2024). Pore-water samples were head-spaced with helium gas and analysed using VICI Trace Gas Analyser with the detection limit of 0.05 ppmv.

**Figure S2. Nitrogen-cycling metabolic capacity of microbial communities in permeable sediments.** Maximum-likelihood phylogenomic tree showing nitrogen cycling metabolic gene inventories of 95 bacterial isolates and 245 bacterial MAGs generated from this study and acquired from [1,2]. The tree was built with 1,000 ultrafast bootstrap replicates using the LG+F+I+G4 model via IQ-TREE. Tree branches were coloured-index to show phylum level taxonomy of bacterial isolates and MAGs. Key metabolic genes for denitrification were highlighted using coloured rings. Specific isolates were selected for further analysis, including culture-based activity and kinetic measurements (asterisks

**References**

1.	Chen YJ, Leung PM, Wood JL *et al.* Metabolic flexibility allows bacterial habitat generalists to become dominant in a frequently disturbed ecosystem. *ISME J* 2021;**15**(10):2986–3004. https://doi.org/10.1038/s41396-021-00988-w.

2.	Chen YJ, Leung PM, Cook PLM *et al.* Hydrodynamic disturbance controls microbial community assembly and biogeochemical processes in coastal sediments. *ISME J* 2022;**16**(3):750–63. https://doi.org/10.1038/s41396-021-01111-9.
